# Outcomes of COVID-19 Infection in People Previously Vaccinated Against Influenza: Population-Based Cohort Study Using Primary Health Care Electronic Records

**DOI:** 10.2196/36712

**Published:** 2022-11-11

**Authors:** Maria Giner-Soriano, Vanessa de Dios, Dan Ouchi, Carles Vilaplana-Carnerero, Mònica Monteagudo, Rosa Morros

**Affiliations:** 1 Fundació Institut Universitari per a la Recerca a l'Atenció Primària de Salut Jordi Gol i Gurina (IDIAPJGol) Barcelona Spain; 2 Universitat Autònoma de Barcelona Bellaterra (Cerdanyola del Vallès) Spain; 3 Department of Clinical Pharmacology, Medicines Area Hospital Clinic of Barcelona Barcelona Spain; 4 Plataforma Spanish Clinical Research Network Unidad de Investigación Clínica Fundació Institut Universitari per a la Recerca a l'Atenció Primària de Salut Jordi Gol i Gurina (IDIAPJGol) Barcelona Spain; 5 Institut Català de la Salut Barcelona Spain

**Keywords:** SARS-CoV-2, COVID-19, influenza vaccines, pneumonia, electronic health records, primary health care, vaccination, public health, cohort study, epidemiology, eHeatlh, health outcome, mortality

## Abstract

**Background:**

A possible link between influenza immunization and susceptibility to the complications of COVID-19 infection has been previously suggested owing to a boost in the immunity against SARS-CoV-2.

**Objective:**

This study aimed to investigate whether individuals with COVID-19 could have benefited from vaccination against influenza. We hypothesized that the immunity resulting from the previous influenza vaccination would boost part of the immunity against SARS-CoV-2.

**Methods:**

We performed a population-based cohort study including all patients with COVID-19 with registered entries in the primary health care (PHC) electronic records during the first wave of the COVID-19 pandemic (March 1 to June 30, 2020) in Catalonia, Spain. We compared individuals who took an influenza vaccine before being infected with COVID-19, with those who had not taken one. Data were obtained from Information System for Research in Primary Care, capturing PHC information of 5.8 million people from Catalonia. The main outcomes assessed during follow-up were a diagnosis of pneumonia, hospital admission, and mortality.

**Results:**

We included 309,039 individuals with COVID-19 and compared them on the basis of their influenza immunization status, with 114,181 (36.9%) having been vaccinated at least once and 194,858 (63.1%) having never been vaccinated. In total, 21,721 (19%) vaccinated individuals and 11,000 (5.7%) unvaccinated individuals had at least one of their outcomes assessed. Those vaccinated against influenza at any time (odds ratio [OR] 1.14, 95% CI 1.10-1.19), recently (OR 1.13, 95% CI 1.10-1.18), or recurrently (OR 1.10, 95% CI 1.05-1.15) before being infected with COVID-19 had a higher risk of presenting at least one of the outcomes than did unvaccinated individuals. When we excluded people living in long-term care facilities, the results were similar.

**Conclusions:**

We could not establish a protective role of the immunity conferred by the influenza vaccine on the outcomes of COVID-19 infection, as the risk of COVID-19 complications was higher in vaccinated than in unvaccinated individuals. Our results correspond to the first wave of the COVID-19 pandemic, where more complications and mortalities due to COVID-19 had occurred. Despite that, our study adds more evidence for the analysis of a possible link between the quality of immunity and COVID-19 outcomes, particularly in the PHC setting.

## Introduction

COVID-19 is caused by SARS-CoV-2, a novel coronavirus that emerged in China in 2019, which became the primary agent of a new pandemic that rapidly spread worldwide [1], with an average global infection fatality rate of approximately 0.15%, depending on the data analyzed [2]. SARS-CoV-2 mainly affects the respiratory tract and uses surface proteins in order to infect the host [3].

Although new variants of SARS-CoV-2 have emerged since December 2020, the coronavirus’ genome is composed of RNA and depends on the RNA polymerase to generate its proteins, with a mechanism of error correction that results in a lower mutation rate than the influenza virus [4]. This low mutation rate may suggest that the vaccines developed against SARS-CoV-2, as well as the immunity generated in those patients who were infected, could represent a long-lasting immunity [5,6].

COVID-19, similar to influenza A and B, is caused by RNA virus and produce similar symptoms. The influenza virus needs the hemagglutinin and neuraminidase surface proteins to infect cells, whereas SARS-CoV-2 needs the S protein [5]. Previous in vitro and animal studies suggest an induction pathway of indirect etiological immunity between the influenza vaccine and SARS-CoV-2. Animal models suggest that some influenza subtypes might lead to regulation of the angiotensin-converting enzyme-2, with protective properties against SARS [7]. An unspecific effect of infection and vaccination on the immune system and susceptibility to other infections has also been reported, albeit with discordant data [8-10]. Some modeling studies have suggested a possible association between influenza immunization and COVID-19 [11-14].

A study conducted in Australia assessed the cellular and humoral immune responses during and after disease occurrence in a patient with a mild COVID-19 infection. They found that the immune response in different cell types is associated with clinical recovery. These results are coincident with similar findings among patients with influenza reported by the same authors [15,16].

Other studies observed differences in the susceptibility to COVID-19 in children of different ages with a lower infection rate than that in adults and older individuals [17]. Although the mechanism underlying these differences in severity and susceptibility is unclear, a possible explanation might be the difference in the quantity and quality of the immune function determined by the history of infections and the recent vaccines administered [18].

Consequently, a link between the quality of the immunity and recovery from COVID-19 may exist. Thus, we hypothesized that the immunity resulting from the previous influenza vaccination would boost part of the immunity against SARS-CoV-2, and we aimed to investigate whether individuals with COVID-19 could have benefited from vaccination against influenza.

## Methods

### Study Design

We performed a population-based cohort study including all adults with COVID-19 in Catalonia, Spain, who were registered as confirmed cases (through the polymerase chain reaction [PCR]) or as probable cases (not confirmed through PCR but with International Classification of Diseases (ICD)-10 codes registered that are compatible with COVID-19) in the primary health care (PHC) system. All individuals with COVID-19 were diagnosed from the pandemic’s onset (March 2020) to June 30, 2020. Participants were compared on the basis of their influenza vaccination status between those having received the influenza vaccine before having COVID-19 (vaccinated in the previous influenza seasonal campaign in 2019-2020 or before) [19] with those who were not vaccinated.

### Data Source

Our data source is the Information System for Research in Primary Care [20], which captures clinical information of approximately 5.8 million people from Catalonia, Spain (approximately 80% of the Catalan population). This information is pseudonymized, having originated from different data sources: (1) electronic health records in PHC system of the Catalan Health Institute, including sociodemographic characteristics, residents in nursing homes or long-term care facilities (LTCFs), comorbidities registered as ICD-10 codes [21], specialist referrals, clinical parameters, toxic habits, sickness leave, date of death, laboratory test data, and drug prescriptions issued in the PHC system, registered in accordance with the anatomical therapeutic chemical classification system [22]; (2) pharmacy invoice data corresponding to the PHC drug prescriptions; (3) database of diagnoses upon hospital discharge [23]; and (4) COVID-19 data from the Catalan Agency of Health Quality and Evaluation (AQuAS) [24].

### COVID-19 Classification

Participants were classified in accordance with the following criteria: *confirmed cases* are those with a confirmed COVID-19 diagnosis record, positive PCR outcome, or a positive serology test result. Those with an unconfirmed diagnosis or test (possible or unclear) along with any individual with a record of hospitalization, pneumonia, or death related to COVID-19 were considered *probable cases*. During the first wave of the COVID-19 pandemic in Catalonia, PCR tests were not routinely conducted for all individuals with compatible symptoms owing to the unavailability of laboratory kits to carry out the tests. Thus, we needed to capture those patients with a possible diagnosis of COVID-19, such as those admitted to hospital with pneumonia or other COVID-19 symptoms, who were not tested. We designed an algorithm to classify patients as “COVID possible” when a test result was unavailable along with registered entries from different databases: PCR tests or serology tests conducted in different settings, discharge diagnoses of pneumonia from Catalan hospitals or from emergency departments, and ICD-10 diagnoses related to COVID-19 coded in PHC. The date of COVID-19 diagnosis was set to be the first of all records used per patient. To guarantee that our algorithm is not far from the Catalan population, the resulting cohort was compared to the official COVID-19 cases reported by the AQuAS during the pandemic [24].

### Influenza Immunization

Patients were classified as having taken the influenza vaccine if they had been vaccinated at any time before having COVID-19, and grouped in accordance with the seasonal vaccination campaign: the immediate previous campaign (2019-2020) or other vaccination campaigns (2018-2019 and before) [19,25].

### Variables

At baseline, the following variables were captured: sex, age, geographical area, MEDEA (Mortalidad en áreas pequeñas Españolas y Desigualdades Económicas y Ambientales [Mortality in small Spanish areas and economic and environmental inequalities]) socioeconomic index (deprivation index based on 5 indicators of socioeconomic position; it helps analyze health inequity, and higher the MEDEA socioeconomic index, worse the deprivation) [26], BMI, residence in nursing homes, smoking habits, comorbidities, and taking influenza vaccines and pneumococcal and tuberculosis vaccines.

The main outcomes assessed during follow-up (up to June 2020) were at least one of the following variables: diagnosis of pneumonia, hospital admission, and mortality. The risk of these events was analyzed in those people who had been vaccinated against influenza at any time before having COVID-19, in those who were recently vaccinated (campaign of 2019-2020), and in those systematically vaccinated (who had been vaccinated at least during 3 different campaigns). We analyzed the same outcomes excluding those of people living in LTCFs, where vaccination is nearly universal in our country [27].

### Statistical Analysis

Quantitative variables were described as mean (SD) values, whereas categorical variables were described as the proportion of vaccinated and unvaccinated individuals. Univariate analyses were based on the Student *t* test or chi-square test depending on the variable.

For each outcome, we fitted a logistic regression model to estimate an odds ratio (OR) comparing the prevalence of each outcome among individuals given the influenza vaccine to that of unvaccinated individuals. The logistic model was fitted along with other covariables such as smoking habits, age, comorbidities (asthma, autoimmune disorders, prior cerebrovascular disease, chronic kidney disease, chronic pulmonary obstructive disease, diabetes, heart failure, hypertension, ischemic heart disease, mental-behavioral disorders, obesity, organ transplant, and other respiratory diseases), concomitant drugs, and previous vaccines (pneumococcal and tuberculosis). As a sensitivity analysis, we conducted the same analysis on a matched population. Individuals vaccinated against influenza and unvaccinated controls were matched 1:2 in accordance with their age and gender at the time of infection or on an index date, and the reported ORs were obtained by fitting a conditional logistic regression model (clogit) accounting for matched pairs and adjusted using the same covariables as in the logistic model. We used the Wald test on the fitted coefficient to determine whether the log-odds were significantly different from 0 at a threshold of .05. All analyses were performed in R (version 4.1.0 or above; The R Foundation).

### Ethical Considerations

The study protocol was approved by the Research Ethics Committee of Institut Universitari d’Investigació en Atenció Primària (June 3, 2020). This is a database research study that has been conducted in accordance with the tenets of the Declaration of Helsinki (Fortaleza, Brazil 2013) and does not require consent from the study participants for the purpose of publication. The need for consent was waived by the Research Ethics Committee of Institut Universitari d’Investigació en Atenció Primària as it is deemed unnecessary according to the European legislation (Regulation [EU] 2016/679).

## Results

We included 309,039 individuals with COVID-19 during the first wave of the pandemic in accordance with their influenza immunization status (Table 1, Multimedia Appendix 1); 114,181 (36.9%) participants had received the influenza vaccine at least once before having COVID-19 and 194,858 (63.1%) had not been vaccinated, with more women in both groups, especially in the vaccinated cohort (61.0% women vs 39.0% men). The mean age was higher for vaccinated individuals (64.3 years, with 52.3% of them being older than 65 years). Vaccinated individuals had more comorbidities than unvaccinated individuals.

Of those who received the influenza vaccine, 66,611 (58.3%) had been recently vaccinated (2019-2020) and 75,311 (66%) had been systematically vaccinated against influenza at least during 3 different years (Table 2).

Of the participants with COVID-19, 11,000 (5.7%) unvaccinated and 21,721 (19%) vaccinated participants presented at least one of the following events: hospital admission, pneumonia, or death. For those who received the influenza vaccine at any time before having COVID-19, the risks of hospitalization (adjusted OR 1.14, 95% CI 1.10-1.19) and death (OR 1.32, 1.23-1.42) were higher than those among unvaccinated participants. For the recently vaccinated participants, the risk was higher for hospitalization (OR 1.16, 95% CI 1.1-1.23), pneumonia (OR 1.12, 95% CI 1.02-1.23), and death (OR 1.14, 95% CI 1.04-1.24). For people with recurrent vaccination, the risk of the 3 outcomes was also higher that among unvaccinated participants (OR 1.07, 1.16, and 1.24, respectively; Table 3). We have also analyzed the results in a matched population of vaccinated versus unvaccinated participants, revealing a higher risk of pneumonia and mortality, with an adjusted OR of 1.11 (95% CI 1.01-1.23) and 1.28 (95% CI 1.07-1.53), respectively (Multimedia Appendix 2).

**Figure 1:**
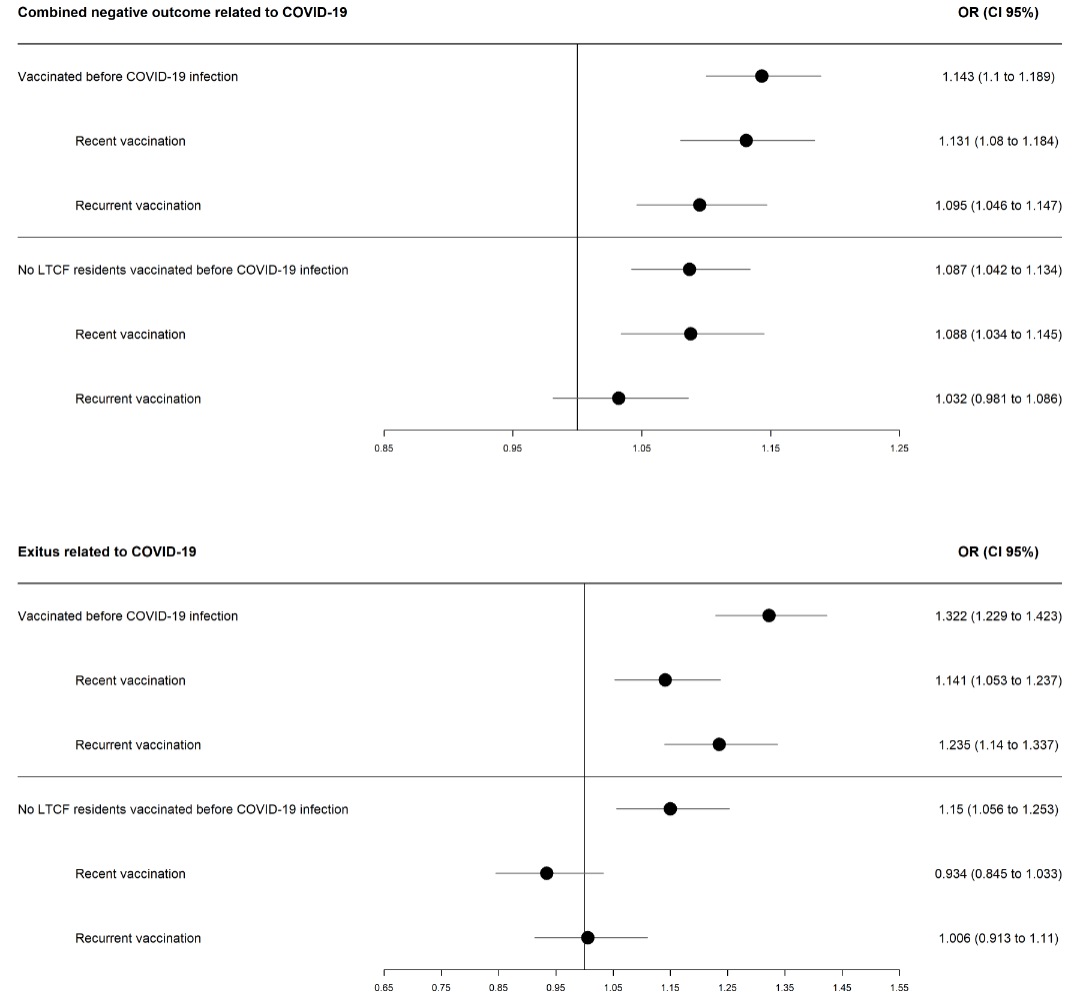
Risk of death and of combined COVID-19 complications in all the vaccinated population and excluding people living in long-term care facilities (LTCF).

The risks of the outcomes based on influenza vaccination status and excluding those patients living in LTCFs are shown in Figure 1. For non-LTCF residents, the results are similar to those for the whole population, except that there was no significant increase in mortality (OR 0.93, 95% CI 0.85-1.03).

**Table 1:** Sociodemographic and clinical characteristics of the study population (N=309,039).

Characteristics			Overall		Not vaccinated against influenza (n=194,858)		Vaccinated against influenza at least once before having COVID-19 (n=114,181)		*P* value	
**COVID-19 status, n (%)**										<.001
	Confirmed	164,557 (53.2)		105,788 (54.3)		58,769 (51.5)				
	Possible	144,482 (46.8)		89,070 (45.7)		55,412 (48.5)				
**Gender, n (%)**										<.001
	Female	173,071 (56.0)		103,413 (53.1)		69,658 (61.0)				
	Male	135,968 (44.0)		91,445 (46.9)		44,523 (39.0)				
Age (years), mean (SD)* *			49.3 (22.3)		40.6 (17.5)		64.3 (21.7)		<.001	
**Age groups (years), n (%)**										<.001
	≤40	108,950 (35.3)		90,894 (46.6)		18,056 (15.8)				
	41-65	129,576 (41.9)		93,116 (47.8)		36,460 (31.9)				
	>65	70,513 (22.8)		10,848 (5.6)		59,665 (52.3)				
Smoker status, n (%)* *			119,554 (38.7)		72,806 (37.4)		46,748 (40.9)		<.001	
Having obesity, n (%)			78,882 (25.5)		36,973 (19.0)		41,909 (36.7)		<.001	
Residents of long-term care facilities, n (%)* *			28,360 (9.2)		3146 (1.6)		25,214 (22.1)		<.001	
**Geographical information (MEDEA)**										<.001
	Unknown	278 (0.1)		201 (0.1)		77 (0.1)				
	Urban	252,014 (81.5)		159,859 (82.0)		92,155 (80.7)				
	Rural	56,747 (18.4)		34,798 (17.9)		21,949 (19.2)				
**Comorbidities, n (%)**										<.001
	Asthma	22,734 (7.4)		9029 (4.6)		13,705 (12.0)				
	Autoimmune disorders	30,783 (10.0)		14,005 (7.2)		16,778 (14.7)				
	Cancer	23,600 (7.6)		6832 (3.5)		16,768 (14.7)				
	Cerebrovascular disease	6937 (2.2)		1053 (0.5)		5884 (5.2)				
	Chronic kidney disease	18,450 (6.0)		2088 (1.1)		16,362 (14.3)				
	Chronic obstructive pulmonary disease	21,771 (7.0)		6155 (3.2)		15,616 (13.7)				
	Diabetes	30,513 (9.9)		5886 (3.0)		24,627 (21.6)				
	Heart failure	8307 (2.7)		693 (0.4)		7614 (6.7)				
	Hypertension	75,346 (24.4)		21,624 (11.1)		53,722 (47.0)				
	Ischemic heart disease	10,049 (3.3)		1837 (0.9)		8212 (7.2)				
	Mental-behavioral disorders	9010 (2.9)		685 (0.4)		8325 (7.3)				
	Organ transplant	893 (0.3)		213 (0.1)		680 (0.6)				
	Other respiratory diseases	16,476 (5.3)		6407 (3.3)		10,069 (8.8)				
**Other vaccines, n (%)**										<.001
	Pneumococcal	78,104 (25.3)		17,617 (9.0)		60,487 (53.0)				
	Tuberculosis	2974 (1.0)		2412 (1.2)		562 (0.5)				

**Table 2:** Taking influenza vaccines before having COVID-19.

		Vaccinated before having COVID-19 (n=114,181)
**Campaign of 2019-2020 (recent immunization), n (%)**		66,611 (58.3)
	Days from vaccination to infection, median (IQR)	146.0 (127.0-169.0)
**Campaign of 2018-2019, n (%) **		60,161 (52.7)
	Days from vaccination to infection, median (IQR)	515.0 (495.0-539.0)
**Campaign of 2017-2018 or before, n (%)**		102,235 (89.5)
	Days from vaccination to infection, median (IQR)	931.0 (875.0-2018.0)
**Campaigns during which participants were vaccinated before having COVID-19, n (%)**		
	1	26,786 (23.5)
	2	12,084 (10.6)
	≥3 (recurrent immunization)	75,311 (66.0)
	3	7931 (6.9)
	4-5	11,146 (9.8)
	6-10	18,945 (16.6)
	>10	37,289 (32.7)

**Table 3:** Logistic regression model of COVID-19 outcomes based on influenza immunization status.

Any vaccination			Influenza immunization status prior to having COVID-19, n (%)				Multivariable logistic model^a^		
			Unvaccinated (n=194,858)		Vaccinated (n=114,181)		Adjusted odds ratio (95% CI)		*P* value
≥1 outcome			11,000 (5.7)		21,721 (19.0)		1.14 (1.10-1.19)		<.001
Hospitalization			7848 (4.0)		10,592 (9.3)		1.10 (1.05-1.15)		<.001
Pneumonia			3011 (1.6)		2740 (2.4)		1.08 (1.00-1.16)		.07
Death			1899 (0.97)		11,835 (10.4)		1.32 (1.23-1.42)		<.001
**Recent vaccination (with 66,611 vaccinated participants)**									
	≥1 outcome	11,000 (5.7)		15,129 (22.7)		1.13 (1.10-1.18)		<.001	
	Hospitalization	7848 (4.0)		7009 (10.5)		1.16 (1.10-1.23)		<.001	
	Pneumonia	3011 (1.6)		1731 (2.6)		1.12 (1.02-1.23)		.02	
	Death	1899 (0.97)		8800 (13.2)		1.14 (1.05-1.24)		.001	
**Recurrent vaccination (with 75,311 vaccinated participants)**									
	≥1 outcome	11,000 (5.7)		17,798 (23.6)		1.10 (1.05-1.15)		<.001	
	Hospitalization	7848 (4.0)		8122 (10.8)		1.07 (1.02-1.14)		.01	
	Pneumonia	3011 (1.6)		1942 (2.6)		1.16 (1.06-1.27)		.002	
	Death	1899 (0.97)		10,561 (14.0)		1.24 (1.14-1.34)		<.001	

^a^A logistic regression model adjusted with the following relevant covariables was fitted: smoking habits, age, comorbidities (asthma, autoimmune disorders, prior cerebrovascular disease, chronic kidney disease, chronic pulmonary obstructive disease, diabetes, heart failure, hypertension, ischemic heart disease, mental-behavioral disorders, obesity, organ transplant, and other respiratory diseases), co-medication, and previous vaccines (pneumococcal and tuberculosis).

## Discussion

### Principal Findings

We analyzed the negative outcomes among people with COVID-19 (N=309,039) and compared those who had received the influenza vaccine with those who were never vaccinated. Those who received the vaccine any time before having COVID-19 were at a higher risk of complications than those who were unvaccinated. We obtained similar results for those who were recently vaccinated (2019-2020 campaign) and for those who were systematically vaccinated (at least 3 years), and the same comparisons were carried out after excluding individuals living in LTCFs. We also obtained similar results on matching vaccinated and unvaccinated individuals. Thus, we did not find a possible link between receiving the influenza vaccine and presenting better clinical outcomes after a COVID-19 infection.

### Comparison With Prior Work

Some researchers have studied this possible association. Massoudi and Mohit [28] conducted a study in a hospital in Iran including health care workers, with 80 of them COVID-19 cases confirmed through PCR or on the basis of their symptoms, and 181 of them were controls. They concluded that individuals who were confirmed cases were less likely to have received the 2019 influenza vaccine (OR 0.04, 95% CI 0.01-0.14), suggesting a protective association between the influenza vaccine and COVID-19. Their study had several limitations, such as the lack of availability of COVID-19 test kits or the samples limited to the workers of a single hospital [28].

Candelli et al [29] assessed 602 patients with COVID-19 enrolled at the emergency department in a hospital in Italy, of whom 24.9% had been previously vaccinated against influenza. They found that influenza immunization was independently associated with a lower risk of death at 60 days (OR 0.20, 95% CI 0.08-0.51), but not with a reduced need of endotracheal intubation (OR 0.73, 95% CI 0.35-1.56) [29].

A study conducted in Brazil [30] included 92,664 confirmed cases of COVID-19, of whom 31.1% had been recently vaccinated against influenza. They found that the vaccinated individuals were at a lower risk of needing intensive care for COVID-19 (OR 0.92, 95% CI 0.86-0.99), a lower risk of needing respiratory support (OR 0.81, 95% CI 0.74-0.88), and lower odds of mortality (OR 0.82, 95% CI 0.75-0.89) [30].

In a systematic review [31] including 12 studies, the authors examined whether influenza vaccination affects the risk of being infected with SARS-CoV-2 and the risk of complicated illness or poor outcomes among patients with COVID-19, all of whom having been confirmed cases through PCR testing. They concluded that influenza vaccination is unlikely to be associated with an increase in the risk of COVID-19 infection or severity and the risk of associated death [31].

There are reports from some countries with high influenza vaccination rates and high incidences of COVID-19 and mortality [32,33]. For instance, Kline et al [33] compared people vaccinated against influenza with unvaccinated individuals admitted to hospital for COVID-19, and they found no differences in the rate of admission to the intensive care unit, intubation, or other complications [33]. Our results follow these same trends in a cohort of the general population attended to in the PHC system and not only hospitalized patients.

### Limitations

We need to consider that our results correspond to the first wave of the COVID-19 pandemic, when there were more negative outcomes and mortalities due to COVID-19 than in the subsequent waves in our setting; thus, this higher statistical power allowed us to detect differences. Furthermore, in subsequent waves, more confounders might have been present, such as COVID-19 vaccination or effects of the different SARS-CoV-2 variants, making it more difficult to manage their potential effect in the analysis of the outcomes of the infection.

We also need to bear in mind that the target population for the influenza vaccine in our country are people older than 60 years, individuals with chronic comorbidities or immunodeficiency, and health care workers among others [34], some of them being at a high risk of COVID-19 complications, which is why confounding variables were used to adjust the logistic regression model [35]. Nevertheless, estimates of the effectiveness of the influenza vaccine have been frequently confounded, indicating that a different approach should be used with alternative study designs, different from the typical methods used to study drug exposure [36-38].

Among other limitations of our study is the reliability of the COVID-19 diagnoses; we included individuals without a confirmed result, as during the first wave of the pandemic in our setting, PCR tests were not always performed. This limitation has been described in other studies including those conducted at the beginning of the pandemic when diagnostic tests for COVID-19 were not widely available and clinical algorithms were used to assess COVID-19 diagnoses [39]. We compared our number of COVID-19 cases with the official COVID-19 case numbers provided by the AQuAS during the pandemic [24]. Another limitation is the lack of hospital information: we could not capture ICU admissions, ventilation, or treatments administered upon admission, which clearly have an influence on the prognosis and outcomes of COVID-19. Finally, we have not conducted any subgroup analysis that could have indicated any condition potentially resulting in any benefit or harm from influenza vaccination.

### Conclusions

In conclusion, we were not able to establish a protective role of the immunity conferred by the influenza vaccine on the outcomes of COVID-19 infection. Nonetheless, our study adds more evidence to the analysis of the possible link between the quality of the conferred immunity and outcomes of COVID-19 infection, and it has some strengths, such as the large cohort size, its representativeness with respect to the general population, and the completeness of its sociodemographic data. We have already highlighted that our cohort comprises individuals who received care from the PHC system; hence, we have estimated the risk of complications for a different population from the hospitalized individuals who are usually assessed in multiple studies.

## Appendix

Multimedia Appendix 1 Baseline characteristics of the population included by gender.

Multimedia Appendix 2 Conditional logistic regression model for the age and gender matched population. Matching performed in patients ≤ 65 years old to correct for age distribution.

## Abbreviations

AQuAS: Catalan Agency of Health Quality and Evaluation
ICD-10: International Classification of Diseases, version 10
LTCF: long-term care facility
MEDEA: Mortalidad en áreas pequeñas Españolas y Desigualdades Económicas y Ambientales (Mortality in small Spanish areas and economic and environmental inequalities)
OR: odds ratio
PCR: polymerase chain reaction
PHC: primary health care
