# Supplementary material for: Outcomes of COVID-19 Infection in People Previously Vaccinated Against Influenza: Population-Based Cohort Study Using Primary Health Care Electronic Records
Source: JMIR Public Health Surveill. 2022 Nov 11;8(11):e36712. doi: 10.2196/36712 (PMC9662290; doi:10.2196/36712)
Supplement: Multimedia Appendix 1 [file publichealth_v8i11e36712_app1.docx]

| **Supplementary table 1. Baseline characteristics of the population included by gender** |  |  |
| --- | --- | --- |

|  |  |  | **Female** | |  |  | **Male** | |  |
| --- | --- | --- | --- | --- | --- | --- | --- | --- | --- |
|  |  | **Overall** | **Non-vaccinated against flu** | **Flu-vaccinated at least once before COVID-19** | **P-value** | **Overall** | **Non-vaccinated against flu** | **Flu-vaccinated at least once before COVID-19** | **P-value** |
| **N (%)** |  | 173071 | 103413 | 69658 |  | 135968 | 91445 | 44523 |  |
| **COVID-19 status** | Confirmed | 94315 (54.5) | 57514 (55.6) | 36801 (52.8) | <0.001 | 70242 (51.7) | 48274 (52.8) | 21968 (49.3) | <0.001 |
|  | Possible | 78756 (45.5) | 45899 (44.4) | 32857 (47.2) |  | 65726 (48.3) | 43171 (47.2) | 22555 (50.7) |  |
| **Age, mean (SD)** |  | 50.50 (22.55) | 41.11 (17.37) | 64.45 (22.14) | <0.001 | 47.80 (21.89) | 39.93 (17.64) | 63.97 (20.86) | <0.001 |
|  | ≤40 | 59273 (34.2) | 47340 (45.8) | 11933 (17.1) | <0.001 | 49677 (36.5) | 43554 (47.6) | 6123 (13.8) | <0.001 |
|  | 41-65 | 72142 (41.7) | 50141 (48.5) | 22001 (31.6) |  | 57434 (42.2) | 42975 (47.0) | 14459 (32.5) |  |
|  | >65 | 41656 (24.1) | 5932 (5.7) | 35724 (51.3) |  | 28857 (21.2) | 4916 (5.4) | 23941 (53.8) |  |
| **Smoker** |  | 57038 (33.0) | 36174 (35.0) | 20864 (30.0) | <0.001 | 62516 (46.0) | 36632 (40.1) | 25884 (58.1) | <0.001 |
| **Obesity** |  | 46677 (27.0) | 20602 (19.9) | 26075 (37.4) | <0.001 | 32205 (23.7) | 16371 (17.9) | 15834 (35.6) | <0.001 |
| **LTCF resident** |  | 20037 (11.6) | 2366 (2.3) | 17671 (25.4) | <0.001 | 8323 (6.1) | 780 (0.9) | 7543 (16.9) | <0.001 |
| **Geographical information (MEDEA)** | Unknown | 150 (0.1) | 102 (0.1) | 48 (0.1) | <0.001 | 128 (0.1) | 99 (0.1) | 29 (0.1) | <0.001 |
|  | Urban | 31353 (18.1) | 18092 (17.5) | 13261 (19.0) |  | 25394 (18.7) | 16706 (18.3) | 8688 (19.5) |  |
|  | Rural | 141568 (81.8) | 85219 (82.4) | 56349 (80.9) |  | 110446 (81.2) | 74640 (81.6) | 35806 (80.4) |  |
| **Comorbidities** | Asthma | 14238 (8.2) | 5129 (5.0) | 9109 (13.1) | <0.001 | 8496 (6.2) | 3900 (4.3) | 4596 (10.3) | <0.001 |
|  | Autoimmune disorders | 25312 (14.6) | 11633 (11.2) | 13679 (19.6) | <0.001 | 5471 (4.0) | 2372 (2.6) | 3099 (7.0) | <0.001 |
|  | Cancer | 12333 (7.1) | 3894 (3.8) | 8439 (12.1) | <0.001 | 11267 (8.3) | 2938 (3.2) | 8329 (18.7) | <0.001 |
|  | Cerebrovascular disease | 3637 (2.1) | 490 (0.5) | 3147 (4.5) | <0.001 | 3300 (2.4) | 563 (0.6) | 2737 (6.1) | <0.001 |
|  | Chronic kidney disease | 10812 (6.2) | 1046 (1.0) | 9766 (14.0) | <0.001 | 7638 (5.6) | 1042 (1.1) | 6596 (14.8) | <0.001 |
|  | COPD | 9924 (5.7) | 3148 (3.0) | 6776 (9.7) | <0.001 | 11847 (8.7) | 3007 (3.3) | 8840 (19.9) | <0.001 |
|  | Diabetes | 14744 (8.5) | 2294 (2.2) | 12450 (17.9) | <0.001 | 15769 (11.6) | 3592 (3.9) | 12177 (27.3) | <0.001 |
|  | Heart failure | 4931 (2.8) | 355 (0.3) | 4576 (6.6) | <0.001 | 3376 (2.5) | 338 (0.4) | 3038 (6.8) | <0.001 |
|  | Hypertension | 41273 (23.8) | 9852 (9.5) | 31421 (45.1) | <0.001 | 34073 (25.1) | 11772 (12.9) | 22301 (50.1) | <0.001 |
|  | Ischaemic heart disease | 3584 (2.1) | 467 (0.5) | 3117 (4.5) | <0.001 | 6465 (4.8) | 1370 (1.5) | 5095 (11.4) | <0.001 |
|  | Mental-behavioural disorders | 6247 (3.6) | 424 (0.4) | 5823 (8.4) | <0.001 | 2763 (2.0) | 261 (0.3) | 2502 (5.6) | <0.001 |
|  | Organ transplant | 363 (0.2) | 89 (0.1) | 274 (0.4) | <0.001 | 530 (0.4) | 124 (0.1) | 406 (0.9) | <0.001 |
|  | Other respiratory diseases | 7476 (4.3) | 2618 (2.5) | 4858 (7.0) | <0.001 | 9000 (6.6) | 3789 (4.1) | 5211 (11.7) | <0.001 |
| **Other vaccines** | Pneumococcal | 42999 (24.8) | 8432 (8.2) | 34567 (49.6) | <0.001 | 35105 (25.8) | 9185 (10.0) | 25920 (58.2) | <0.001 |
|  | Tuberculosis | 1688 (1.0) | 1342 (1.3) | 346 (0.5) | <0.001 | 1286 (0.9) | 1070 (1.2) | 216 (0.5) | <0.001 |

SD: standard deviation. LTFC: long-term care facility. MEDEA: socioeconomic index. COPD: chronic obstructive pulmonary disease.
