# Supplementary material for: Outcomes of COVID-19 Infection in People Previously Vaccinated Against Influenza: Population-Based Cohort Study Using Primary Health Care Electronic Records
Source: JMIR Public Health Surveill. 2022 Nov 11;8(11):e36712. doi: 10.2196/36712 (PMC9662290; doi:10.2196/36712)
Supplement: Multimedia Appendix 2 [file publichealth_v8i11e36712_app2.docx]

| **Supplementary table 2. Conditional logistic regression model for the age and gender matched population.** | | | | |  |  |
| --- | --- | --- | --- | --- | --- | --- |
|  | **Influenza immunization status prior to COVID-19 (%)** | | **Univariable logistic regression** | | **Multivariable logistic model*** | |
| **Any vaccination** | **Non-vaccinated (N=95,063)** | **Vaccinated (N=52,453)** | **OR (95% CI)** | **P-value** | **aOR (95% CI)** | **P-value** |
| **≥1 outcome** | 5436 (5.7) | 3805 (7.3) | 1.212 (1.162 to 1.264) | <.001 | 1.027 (0.972 to 1.084) | .343 |
| **Hospitalization** | 4109 (4.3) | 2807 (5.4) | 1.176 (1.12 to 1.235) | <.001 | 0.987 (0.927 to 1.051) | .680 |
| **Pneumonia** | 1634 (1.7) | 1063 (2.0) | 1.166 (1.078 to 1.261) | <.001 | 1.111 (1.007 to 1.226) | .037 |
| **Death** | 563 (0.6) | 565 (1.1) | 1.733 (1.542 to 1.949) | <.001 | 1.279 (1.067 to 1.533) | .008 |
| *Matching performed in patients ≤ 65 years old to correct for age distribution* | | | |  |  |  |
